# OLDER ADULT’S PERCEPTION OF PRIMARY HEALTH CARE REFORM IN A LOW-MIDDLE-INCOME COUNTRY (LMIC): IMPACT ON GERIATRIC CARE

**DOI:** 10.1093/geroni/igac059.2148

**Published:** 2022-12-20

**Authors:** Theresa Abah

**Affiliations:** California State University, Sacramento, Sacramento, California, United States

## Abstract

Health reforms are intended to provide equitable and beneficial care across population groups, it also transforms service delivery. We sought to identify current or potential implementation gaps that divert improvement efforts in service delivery, and practices that prevent holistic approaches in caring for older adults, especially in developing nations. This study examined a major health reform initiative in a LMIC aimed at integrating defragmented health organizations under one management, it also looked at extent to which reform agendas align with primary health care (PHC) principles. Using purposeful random sampling design, this quantitative study obtained participants’ views of care received in 12 sites and 2 states in Nigeria. A total of 218 participants aged 50 years and older in both rural and urban areas. Partial correlations were carried out after accounting for other variables of interest. Findings show there was no significant differences in attributes of primary care received by geography, gender or by state. Although Ondo state scored higher than Federal Capital Territory in previous performance evaluations (66% and 43% in 2015), respondents from both states received only two out of the seven primary care attributes recommended by the World Health Organization. First contact (access to care) and Care continuity, p <.01 and p, 0.5, (d = 0.202 and 0.179) were significant. Overall, our findings suggest that performance improvement at management level dominated reform efforts over service delivery. It is unclear how this aligns with providing older adults with the health care that they need.

